# DeltaRex-G, tumor targeted retrovector encoding a CCNG1 inhibitor, for CAR-T cell therapy induced cytokine release syndrome

**DOI:** 10.3389/fmmed.2024.1461151

**Published:** 2024-09-18

**Authors:** Grace Haroun, Erlinda M. Gordon

**Affiliations:** ^1^ University of California, Los Angeles, Los Angeles, CA, United States; ^2^ Sarcoma Oncology Research Center, Santa Monica CA, Aveni Foundation, Santa Monica, CA, United States

**Keywords:** DeltaRex-G, CAR-T cell therapy, cytokine release syndrome, COVID-19, acute respiratory disease

## Abstract

Cytokine release syndrome is a serious complication of chimeric antigen receptor-T cell therapy and is triggered by excessive secretion of inflammatory cytokines by chimeric T cells which could be fatal. Following an inquiry into the molecular mechanisms orchestrating cytokine release syndrome, we hypothesize that DeltaRex-G, a tumor targeted retrovector encoding a cytocidal CCNG1 inhibitor gene, may be a viable treatment option for corticosteroid-resistant cytokine release syndrome. DeltaRex-G received United States Food and Drug Administration Emergency Use Authorization to treat Covid-19-induced acute respiratory distress syndrome, which is due to hyperactivated immune cells. A brief administration of DeltaRex-G would inhibit a certain proportion of hyperactive chimeric T cells, consequently reducing cytokine release while retaining chimeric T cell efficacy.

## Introduction

Chimeric Antigen Receptor-T (CAR-T) cell therapy is a novel cancer treatment wherein CARs are introduced into a patient’s own harvested T cells and subsequently infused intravenously for the purpose of eradicating cancer (Kalos et al., 2011). Factors that limit the efficacy of CAR-T cell therapy include minimal or exaggerated CAR-T cell proliferation, a dysregulated inflammatory tumor microenvironment (TME) and a high baseline tumor burden (Ventin et al., 2024). Cytokine Release Syndrome (CRS) is a severe, potentially fatal, adverse event that could develop in patients receiving CAR-T cell therapy (Freyer and Porter, 2020). Hypothesis: A brief administration of DeltaRex-G, a tumor targeted retroviral vector encoding a cytocidal mutated cyclin G1 gene, would inhibit only the dividing T cells thus reducing cytokine release by hyperactive CAR-T cells while retaining their antitumor efficacy.

### CAR-T cell therapy

T cells harvested from the patient are modified with chimeric antigen receptors (CARs) engineered to recognize and bind antigens specific to a patient’s cancer (Sermer and Brentjens, 2019; Korell et al., 2022). Engineered CAR-T cells equipped to target and eliminate cancer cells are intravenously infused into the patient who has undergone lymphodepletion. Upon recognizing and binding to the target antigen in cancer cells, the activated CAR-T cells eradicate tumor cells while proliferating simultaneously. CAR-T cell therapy has been proven effective in pediatric and adult acute lymphocytic leukemia, B cell lymphoma, mantle cell lymphoma, and multiple myeloma by targeting the Cluster of Differentiation 19 (CD19) or B cell maturation antigen (BCMA) on these malignant cells (Asmamaw et al., 2022).

Currently, there is only one FDA-approved CAR-T cell therapy for solid tumors. The primary challenge to implementing CAR-T cell therapy for solid tumors is tumor heterogeneity and consequent difficulty in ascertaining which antigen, ideally a mutated oncogene, should be targeted (Qin and Xu, 2022). Moreover, solid tumors are often found in tissues with reduced regenerative capacity compared to the hematopoietic system so CAR-T cell targets must be incredibly precise to preserve the maximum amount of healthy tissue (Qin and Xu, 2022; Xia et al., 2018). Two novel receptors enable inducible CAR expression to enhance tumor specificity and prevent CAR-T cell exhaustion, including Synthetic intramembrane proteolysis receptors (SNIPRs) and Signal neutralization by an inhibitable protease (SNIP) (Qin and Xu, 2022; Zhu et al., 2022; Labanieh et al., 2022). Additionally, stroma in tumors act as physical barriers and the immunosuppressive tumor microenvironment (TME) further prevents optimal CAR-T cell penetration in solid tumors (Asmamaw et al., 2022). Ongoing research has determined that intratumoral injection of CAR-T cells and a hydrogel containing cytokine and CAR-T cells can overcome the physical impediments to solid tumors and improve CAR-T cell cytotoxicity and efficacy while preserving tumor-specificity (Qin and Xu, 2022; Melero et al., 2021; Adusumilli et al., 2014; Grosskopf et al., 2022). There are currently over fifty clinical trials investigating various solid tumor targets for CAR-T cell therapy--Mesothelin, Carcinoembryonic antigen, Claudin18.2, and Cluster of Differentiation 70 (CD70) are the most common antigens in addition to dual CAR targets to increase tumor specificity (A Phase I, 2016; T Cells Armed With Chimeric, 2016; Phase I, 2017; Phase I Study of Autologous, 2018; A First in Human Phase, 2018; A Phase I Investigation of the Safety, 2019; An Open Label, 2019; An Exploratory Study of αPD1, 2020; A Phase I Trial to Assess Safety, 2020; Open, 2020; B7-H3-Specific Chimeric Antigen Receptor Autologous, 2021; Phase I Study of EGFR, 2021; A Single, 2021; A Phase 1 Dose Escalation, 2022; A Phase 1 Study to, 2022; Clinical Trial to Evaluate the, 2022; A Single, 2022; A Phase Ia/Ib, 2022; An Exploratory Study of αPD1, 2022; Open-Label, 2022; Single-center, 2022a; A Phase I Clinical Study, 2022a; A Safety and Efficacy Clinical, 2022; A Phase I Clinical Study, 2022b; GD2/PSMA Bi, 2022; A Phase I Clinical Study, 2022d; Clinical Study of CLDN18, 2022; An Open, 2022; A Phase I Clinical Study, 2022c; Chimeric Antigen Receptor T Lymphocytes CAR-T, 2022; Clinical Study to Evaluate the, 2022; Exploratory Clinical Study of PD, 2022; Single-center, 2022b; FIH and Arm, 2022; Efficacy and Safety of Claudin18, 2022; Phase, 2022; A Phase 1, 2023; A Phase1/phase2 and Single-arm, 2023; Mesothelin/GPC3/GUCY2C Targeted CAR, 2023; A Clinical Study on the, 2023; Exploratory Clinical Trial on the, 2023b; A Study to Evaluate the Safety et al., 2023; Phase I, 2023; Exploratory Clinical Trial on the, 2023a; FTiH, 2023; A Phase I Clinical Study, 2023; A Safety and Efficacy Clinical, 2023; An Exploratory Clinical Study Evaluating, 2023a; Phase I Clinical Study of, 2023; A Seamless Phase 1, 2023; An Exploratory Clinical Study Evaluating, 2023b; A Phase 1 Study of, 2024; An Exploratory Study on the, 2024; Exploratory Study of MSLN, 2024; Exploratory Study on the Treatment, 2024; Brudno and Kochenderfer, 2016; Maude et al., 2014) (A Phase I, 2016; T Cells Armed With Chimeric, 2016; Phase I, 2017; Phase I Study of Autologous, 2018; A First in Human Phase, 2018; A Phase I Investigation of the Safety, 2019; An Open Label, 2019; An Exploratory Study of αPD1, 2020; A Phase I Trial to Assess Safety, 2020; Open, 2020; B7-H3-Specific Chimeric Antigen Receptor Autologous, 2021; Phase I Study of EGFR, 2021; A Single, 2021; A Phase 1 Dose Escalation, 2022; A Phase 1 Study to, 2022; Clinical Trial to Evaluate the, 2022; A Single, 2022; A Phase Ia/Ib, 2022; An Exploratory Study of αPD1, 2022; Open-Label, 2022; Single-center, 2022a; A Phase I Clinical Study, 2022a; A Safety and Efficacy Clinical, 2022; A Phase I Clinical Study, 2022b; GD2/PSMA Bi, 2022; A Phase I Clinical Study, 2022d; Clinical Study of CLDN18, 2022; An Open, 2022; A Phase I Clinical Study, 2022c; Chimeric Antigen Receptor T Lymphocytes CAR-T, 2022; Clinical Study to Evaluate the, 2022; Exploratory Clinical Study of PD, 2022; Single-center, 2022b; FIH and Arm, 2022; Efficacy and Safety of Claudin18, 2022; Phase, 2022; A Phase 1, 2023; A Phase1/phase2 and Single-arm, 2023; Mesothelin/GPC3/GUCY2C Targeted CAR, 2023; A Clinical Study on the, 2023; Exploratory Clinical Trial on the, 2023b; A Study to Evaluate the Safety et al., 2023; Phase I, 2023; Exploratory Clinical Trial on the, 2023a; FTiH, 2023; A Phase I Clinical Study, 2023; A Safety and Efficacy Clinical, 2023; An Exploratory Clinical Study Evaluating, 2023a; Phase I Clinical Study of, 2023; A Seamless Phase 1, 2023; An Exploratory Clinical Study Evaluating, 2023b; A Phase 1 Study of, 2024; An Exploratory Study on the, 2024; Exploratory Study of MSLN, 2024; Exploratory Study on the Treatment, 2024; Brudno and Kochenderfer, 2016; Maude et al., 2014)

### Cytokine release syndrome

CAR-T cell therapy stimulates a robust immune response that can cause cytokine release syndrome (CRS) in patients, which can be fatal. CRS begins as a fever and myalgia two to 3 days after CAR-T cell infusion but can progress to capillary leak, hypoxia, hypotension, tachycardia, pulmonary edema, and pleural edema within 2 weeks of treatment (Sermer and Brentjens, 2019). In dire cases, organ failure and death may ensue. The molecular basis for CRS is excessive secretion of cytokines by T cells (Brudno and Kochenderfer, 2016),. One of the core cytokines elevated in CRS patient serum is the inflammatory interleukin-6 (IL-6) produced by monocytes, macrophages, and T-cells (Maude et al., 2014). Patients with high IL-6 levels and large baseline tumor burdens have inflammatory TMEs that prime myeloid cells and macrophages to induce an immune response, and this condition is amplified by CAR-T cell treatment. Elevated IL-6 levels post-treatment have been correlated with diminished response to CAR-T cell therapy and severe CRS (Sermer and Brentjens, 2019). Tocilizumab, an anti-IL-6R antibody, is the current standard of care for mild CRS but corticosteroid therapy like dexamethasone is often required in cases of severe CRS. Recent studies have shown that high dose corticosteroid treatment and early CRS intervention with Tocilizumab and preemptive corticosteroids decreased the risk of severe CRS without adversely affecting CAR-T cell therapy efficacy (Gardner et al., 2019; Liu et al., 2020). Anakirna, an IL-1 receptor antagonist has been identified as another therapeutic for severe CRS (grade 3 or 4) when administered with corticosteroids, and if administered early in combination with Tocilizumab, can prevent CRS (Ferreros and Trapero, 2022; Gazeau et al., 2023). Siltuximab, another anti-IL-6 antibody, has also shown promising results in treating mild CRS (Bajwa et al., 2024). However, these treatments are not universally effective and as such, a demand for a CRS treatment that will not only mitigate the potentially devastating progression of CRS but could also maximize the potency of the administered CAR-T cell therapy persists (Chawla et al., 2022).

### DeltaRex-G for cytokine release syndrome

Originally developed as a cancer drug, DeltaRex-G (formerly named Rexin-G, Mx-dnG1) is a tumor targeted retrovector encoding a cytocidal CCNG1 inhibitor gene which inhibits cyclin G1 expression, and consequently, blocks the cancer cell cycle in G0-G1 phase, aborting the cell cycle and resulting in cell death via the apoptosis-mediated pathway (Morse et al., 2021; Chawla et al., 2019). The membrane gp70 envelope of DeltaRex-G was molecularly engineered to display a signature (SIG) protein-binding decapeptide that recognizes and binds to abnormal anaplastic collagenous SIG proteins in the TME, then fuses and enters via the innate amphotropic Pit2 receptor and inhibits/destroys only highly proliferative cells including cancer cells, neoangiogenic cells and stroma-producing fibroblasts (Morse et al., 2021; Chawla et al., 2019). Phase I/II studies in patients with pancreatic adenocarcinoma, sarcomas, and metastatic breast cancers have established a significant association between DeltaRex-G dosage and tumor control/survival advantage (Chawla et al., 2019; Chawla et al., 2016; Liu et al., 2021). During the COVID-19 pandemic, DeltaRex-G was granted FDA Emergency Use Authorization for severe COVID-19 induced CRS and acute respiratory distress syndrome (ARDS) (Larkin, 2021). CRS from CAR-T cell therapy and COVID-19 develop from excessive stimulation and activation of immune cells and consequent cytokine release resulting in tissue damage.

**FIGURE 1:**
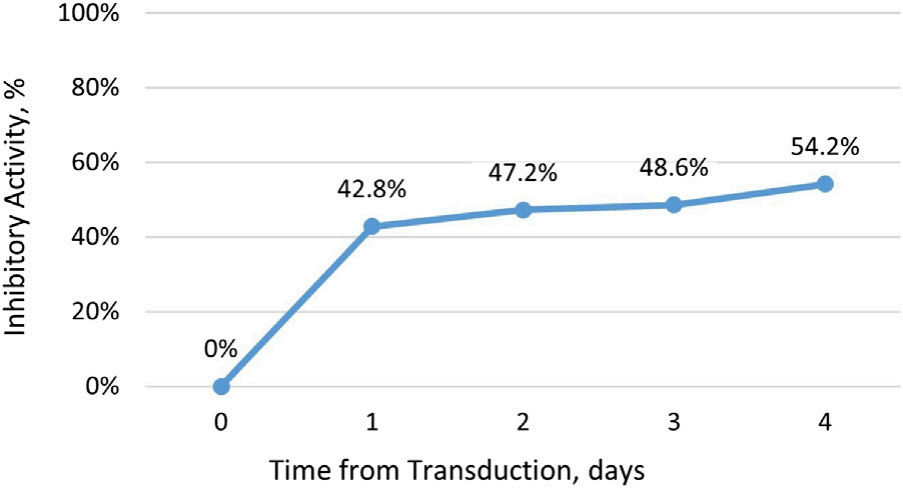
Inhibitory activity of DeltaRex-G in CD4^+^ CD8^+^ T cell cultures. The percent inhibitory activity of DeltaRex-G is plotted on the vertical axis as a function of time (days) from retroviral transduction of CD4^+^ CD8^+^ T cell cultures.

**FIGURE 2:**
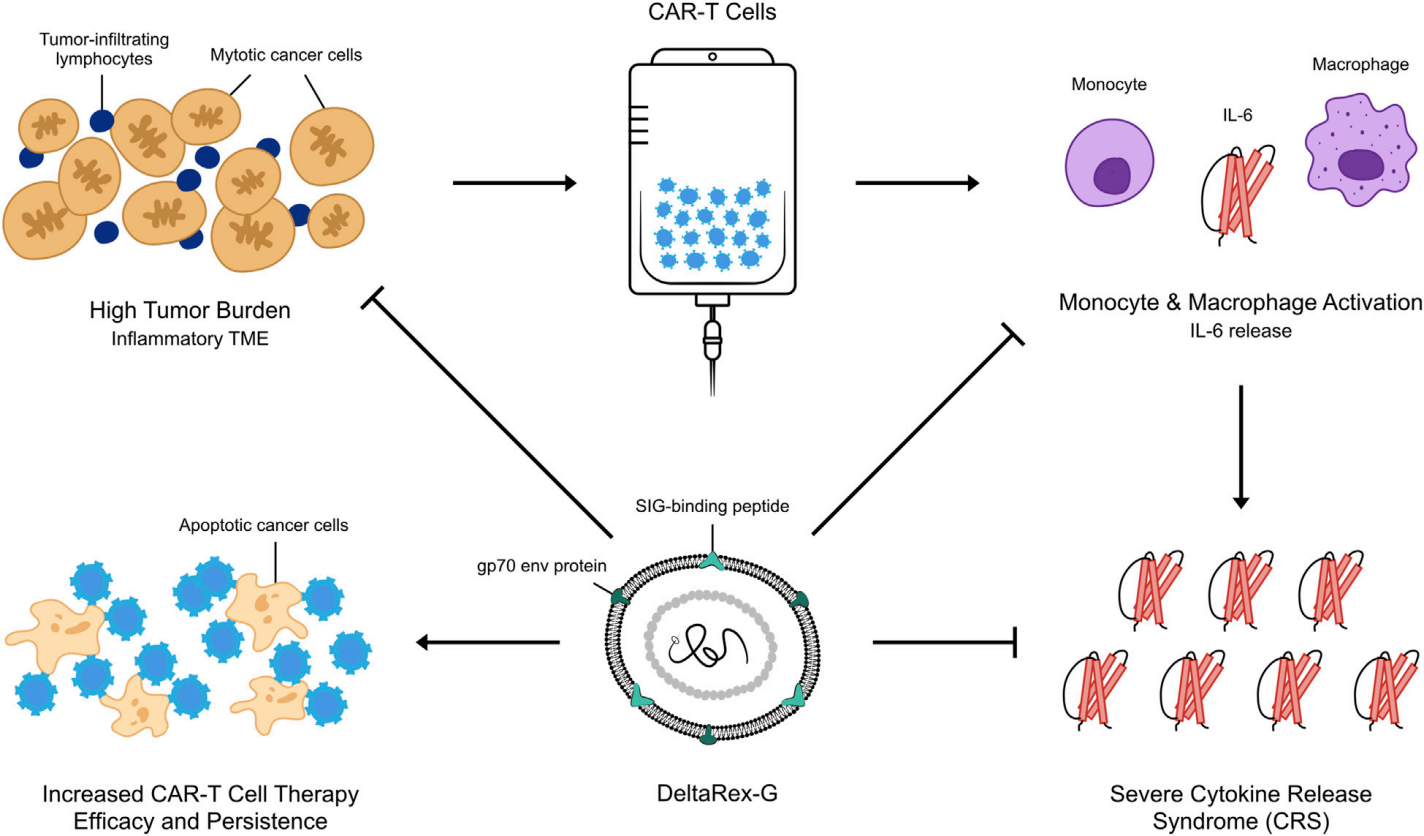
An artist’s illustration of DeltaRex-G mechanism of action in CAR-T cell induced severe CRS. By killing a certain proportion of actively dividing CAR-T cells and cancer cells, the secretion of inflammatory cytokines by chimeric T cells is reduced while retaining the efficacy of remaining CAR-T cells in reducing tumor burden.

Recently, we demonstrated the inhibitory activity of DeltaRex-G in cultures of CD4^+^ CD8^+^ T cells (See Figure 1). This led to the hypothesis that DeltaRex-G could also inhibit the activity of activated CAR-T cells that cause CRS while retaining CAR-T cell efficacy (See Figure 2). The fact that DeltaRex-G (a retroviral based vector) only integrates in the chromosome of rapidly dividing cells, a property that is common in rapidly dividing cancer cells and proliferating CAR-T cells, is the rationale for using DeltaRex-G in CAR-T induced CRS. Further, DeltaRex-G is not immunogenic, so excessive immune responses are not expected to result from DeltaRex-G treatment. In fact, previous studies in cancer patients showed no development of CRS in DeltaRex-G treated patients and is not expected to cause any serious adverse events, including B-cell aplasia and neurotoxicity which are major sequelae of CAR-T cell therapy (Chawla et al., 2022; Chawla et al., 2019; Chawla et al., 2016; Liu et al., 2021; Bruckner et al., 2023).

## Discussion and conclusion

Our hypothesis that a brief administration of DeltaRex-G would reduce the severity of CAR-T cell therapy-induced CRS is supported by the inhibitory activity of DeltaRex-G in transduced CD4 CD8 cell cultures (Figure 1). DeltaRex-G may be used to treat CRS by inhibiting a certain proportion of the proliferative cytokine-releasing immune cells, hence reducing production of IL-6, while retaining the efficacy of unaffected CAR-T cells (Figure 2). Clinical data from cancer patients treated with DeltaRex-G have shown an initial control of tumor growth with eventual tumor shrinkage and attainment of clinical remission after 8 months of DeltaRex-G therapy. Albeit DeltaRex-G has not yet been used to treat CRS, DeltaRex-G has not been reported to cause hematologic nor organ dysfunction in Phase 1 and Phase studies using DeltaRex-G in advanced sarcoma, pancreatic cancer and carcinoma of breast (Chawla et al., 2019; Chawla et al., 2016; Liu et al., 2021; Bruckner et al., 2023). Further no vector neutralizing antibodies have formed with prolonged DeltaRex-G therapy, indicating that DeltaRex-G is not immunogenic (Chawla et al., 2019; Chawla et al., 2016; Liu et al., 2021; Bruckner et al., 2023). Additionally, no delayed adverse events have been reported in long term (>15 years) cancer survivors with DeltaRex-G treatment (Liu et al., 2021). Nevertheless, a phase 1/2 clinical study is warranted to show the safety and inhibitory activity of DeltaRex-G in patients suffering from steroid-resistant cytokine release syndrome following CAR-T cell therapy.

## Data Availability

The original contributions presented in the study are included in the article/supplementary material, further inquiries can be directed to the corresponding author.

## References

[B1] A Study to Evaluate the Safety, TolerabilityEfficacy, Preliminary (2023). Pharmacodynamics and immunogenicity of universal CNK-ut cells in patients with advanced solid tumors. Available at: https://clinicaltrials.gov/study/NCT05976906.

[B2] Adusumilli P. S. Cherkassky L. Villena-Vargas J. Colovos C. Servais E. Plotkin J. (2014). Regional delivery of mesothelin-targeted CAR T cell therapy generates potent and long-lasting CD4-dependent tumor immunity. Sci. Transl. Med. 6 (261), 261ra151. 10.1126/scitranslmed.3010162 PMC4373413 25378643

[B3] An Open (2022). Single-arm clinical study of autologous T cells (CAR-T) targeting B7-H3 chimeric antigen receptor gene in the treatment of patients with advanced gastrointestinal tumors. Available at: https://clinicaltrials.gov/study/NCT05515185.

[B4] An Open Label (2019). “Single/multiple dose exploratory clinical study to evaluate the safety, efficacy, and cytokinetics of autologous humanized anti-claudin18,” in 2 chimeric antigen receptor T cell in advanced solid tumor subjects. Available at: https://clinicaltrials.gov/study/NCT03874897.

[B5] A Phase1/phase2, Single-arm (2023). Open-label study of RD14-01 in patients with advanced solid tumors. Available at: https://clinicaltrials.gov/study/NCT05748938.

[B6] A Phase Ia/Ib (2022). Open-label, single-arm, dose-escalation and expansion study of specific dual-targeting VEGFR1 and PD-L1 CAR-T in cancer patients with pleural or peritoneal metastases. Available at: https://clinicaltrials.gov/study/NCT05477927.

[B7] A Phase I Investigation of the Safety (2019). Tolerability and immunological effects of T Lymphocytes transduced with an anti-lewis Y (LeY) chimeric antigen receptor gene (LeY-CAR-T) in patients with LeY antigen expressing advanced solid tumours. Available at: https://clinicaltrials.gov/study/NCT03851146.

[B8] A Phase I Trial to Assess Safety (2020). Tolerability and anti-tumor activity of autologous T cell modified chimeric antigen receptor (CAR) (CCT303-406) in patients with relapsed or refractory HER2 positive solid tumors. Available at: https://clinicaltrials.gov/study/NCT04511871.

[B9] Asmamaw D. T. Tiruneh G. /M. M. Dessie Terefe G. Tadele Admasu F. Wale Tesega W. Chekol Abebe E. (2022). Current updates on generations, approvals, and clinical trials of CAR T-cell therapy. Hum. Vaccines and Immunother. 18 (6), 2114254. 10.1080/21645515.2022.2114254 PMC9746433 36094837

[B29] An exploratory study of αpd1-MSLN-CAR T cells secreting PD-1 nanobodies for the treatment of MSLN-positive advanced solid tumors (2020). Available at: https://clinicaltrials.gov/study/NCT04503980

[B30] An exploratory study of αpd1-MSLN-CAR T cells secreting PD-1 nanobodies for the treatment of MSLN-positive advanced solid tumors (2022). Available at: https://clinicaltrials.gov/study/NCT05373147

[B35] A First in human phase I trial of binary oncolytic adenovirus in combination with HER2-specific autologous CAR T cells in patients with advanced HER2 positive solid tumors (2018). Available at: https://clinicaltrials.gov/study/NCT03740256

[B18] A clinical study on the safety and preliminary efficacy of IMC008 in the treatment of CLDN18.2- positive advanced solid tumors (2023). Available at: https://clinicaltrials.gov/study/NCT05837299

[B23] An exploratory clinical study evaluating the safety and efficacy of anti-CEA-CAR-T cells injection in patients with CEA+ locally advanced and/or metastatic solid tumors (2023a). Available at: https://clinicaltrials.gov/study/NCT06013111

[B24] An exploratory clinical study evaluating the safety and efficacy of anti-HER2-CAR-T cells injection in patients with HER2+ locally advanced and/or metastatic solid tumors (2023b). Available at: https://clinicaltrials.gov/study/NCT06101082

[B66] A phase I/II study administering peripheral Blood Lymphocytes transduced with a CD70-binding chimeric antigen receptor to patients with CD70-expressing cancers (2016). Available at: https://clinicaltrials.gov/study/NCT02830724

[B71] A safety and efficacy clinical study of CEA-targeted CAR-T therapy for CEA-positive advanced malignant solid tumors (2022). Available at: https://clinicaltrials.gov/study/NCT05415475

[B72] A safety and efficacy clinical study of CEA-targeted CAR-T therapy forCEA-positive advanced/metastatic malignant solid tumors (2023). Available at: https://clinicaltrials.gov/study/NCT06010862

[B73] A seamless phase 1/2 study to evaluate the safety and efficacy of A2B694, an autologous logic-gated Tmod^TM^ CAR T, in Heterozygous HLA-A*02 adults with recurrent unresectable, locally advanced, or metastatic solid tumors that express MSLN and have lost HLA-A*02 expression (2023). Available at: https://clinicaltrials.gov/study/NCT06051695

[B54] A phase 1 dose escalation and expanded cohort study of P-MUC1C-ALLO1 in adult subjects with advanced or metastatic solid tumors (2022). Available at: https://clinicaltrials.gov/study/NCT05239143

[B55] A phase 1 study of FT825/ONO-8250, an off-the-shelf CAR T-cell therapy, with or without monoclonal antibodies (2024). in HER2-Positive or other advanced solid tumors Available at: https://clinicaltrials.gov/study/NCT06241456

[B56] A phase 1 study to assess the safety and efficacy of LYL797, ROR1-targeting CAR T cells (2022). in Adults with relapsed and/or refractory solid-tumor malignancies Available at: https://clinicaltrials.gov/study/NCT05274451

[B57] A phase 1/2 study to evaluate the safety and efficacy of A2B530, an autologous logic-gated Tmod^TM^ chimeric antigen receptor T cell (CAR T), in heterozygous HLA-A*02 adult subjects with recurrent unresectable (2023). Local. Adv. or Metastatic Solid Tumors That Express CEA Have Lost HLA-A*02 Expr. Available at: https://clinicaltrials.gov/study/NCT05736731

[B75] A single-arm, open, exploratory clinical study evaluating the safety and efficacy of EGFR/B7H3 CAR-T in patients with EGFR/B7H3-positive advanced solid tumors (lung and triple-negative breast cancer) (2022). Available at: https://clinicaltrials.gov/study/NCT05341492

[B59] A phase I clinical study of anti-CEA CAR-T therapy in the treatment of CEA-positive advanced malignant solid tumors. (2022a) Available at: https://clinicaltrials.gov/study/NCT05396300

[B60] A phase I clinical study of CD70-targeting CAR-T therapy in the treatment of CD70-positive advanced/metastatic solid tumors (2022b). Available at: https://clinicaltrials.gov/study/NCT05420545

[B61] A phase I clinical study of CD70-targeting CAR-T therapy in the treatment of CD70-positive advanced/metastatic solid tumors. (2022c). Available at: https://clinicaltrials.gov/study/NCT05518253

[B78] A single-center exploratory clinical study to evaluate the safety and efficacy of RD133 in subjects with relapsed or refractory MSLN-positive solid tumors (2021). Available at: https://clinicaltrials.gov/study/NCT05141253

[B63] A phase I clinical study to assess the safety and efficacy of CD70-targeted CAR-T in the treatment of CD70-positive advanced/metastatic solid tumors (2023). Available at: https://clinicaltrials.gov/study/NCT06010875

[B64] A phase I clinical study to assess the safety and tolerability of CD70-targeting CAR-T in the treatment of CD70-positive advanced/metastatic solid tumors (2022d). Available at: https://clinicaltrials.gov/study/NCT05468190

[B10] B7-H3-Specific chimeric antigen receptor autologous T-cell therapy for pediatric patients with solid tumors (3CAR) (2021). Available at: https://clinicaltrials.gov/study/NCT04897321

[B11] Bajwa A. Zhao Q. Geer M. J. Mian A. Lin C. Frame D. (2024). Efficacy of Siltuximab for chimeric antigen receptor T-cell therapy toxicities – a multicenter retrospective Analysis. Transplant. Cell. Ther. 30 (2), S201–S202. 10.1016/j.jtct.2023.12.261

[B12] Bruckner H. W. Chawla S. P. Omelchenko N. Brigham D. A. Gordon E. M. (2023). Phase I-II study using DeltaRex-G, a tumor-targeted retrovector encoding a cyclin G1 inhibitor for metastatic carcinoma of breast. Front. Mol. Med. 3, 1105680. 10.3389/fmmed.2023.1105680 39086675 PMC11285576

[B13] Brudno J. N. Kochenderfer J. N. (2016). Toxicities of chimeric antigen receptor T cells: recognition and management. Blood 127 (26), 3321–3330. 10.1182/blood-2016-04-703751 27207799 PMC4929924

[B14] Chawla S. P. Bruckner H. Morse M. A. Assudani N. Hall F. L. Gordon E. M. (2019). A phase I-II study using rexin-G tumor-targeted retrovector encoding a dominant-negative cyclin G1 inhibitor for advanced pancreatic cancer. Mol. Ther. - Oncolytics 12, 56–67. 10.1016/j.omto.2018.12.005 30705966 PMC6348982

[B15] Chawla S. P. Chawla N. S. Quon D. Chua-Alcala V. Blackwelder W. C. Hall F. L. (2016). An advanced phase 1/2 study using an XC-targeted gene therapy vector for chemotherapy resistant sarcoma. Sarcoma Res. Int. 3 (1), 1024. Available at: https://austinpublishinggroup.com/sarcoma/fulltext/sarcoma-v3-id1024.pdf.

[B16] Chawla S. P. Wong S. Quon D. Moradkhani A. Chua V. S. Brigham D. A. (2022). Three year results of Blessed: expanded access for DeltaRex-G for an intermediate size population with advanced pancreatic cancer and sarcoma (NCT04091295) and individual patient use of DeltaRex-G for solid malignancies (IND# 19130). Front. Mol. Med. 2, 1092286. 10.3389/fmmed.2022.1092286 39086973 PMC11285611

[B17] Chimeric Antigen Receptor T Lymphocytes (CAR-T) (2022). Targeting CEA in the treatment of CEA positive clinical study of advanced malignant solid tumors. Available at: https://clinicaltrials.gov/study/NCT05538195.

[B19] Clinical study to evaluate the safety and efficacy of U87 CART in treatment of advanced solid tumor (2022). Available at: https://clinicaltrials.gov/study/NCT05605197

[B20] Clinical trial to evaluate the safety and efficacy of IM96 CAR-T cells therapy in patients with advanced digestive system neoplasms (2022). Available at: https://clinicaltrials.gov/study/NCT05287165

[B21] Clinical Study of CLDN18 (2022). 2-targeting chimeric antigen receptor-modified autologous T cells in advanced solid tumors with positive CLDN18, 2. Available at: https://clinicaltrials.gov/study/NCT05472857.

[B22] Efficacy and Safety of Claudin18 (2022). 2CAR-T Adv. Pancreat. Cancer Gastric Carcinoma. Available at: https://clinicaltrials.gov/study/NCT05620732.

[B25] Exploratory clinical study of PD-1 knockout targeting MUC1 CAR-T cells (AJMUC1) in the treatment of MUC1-positive advanced breast cancer (2022). Available at: https://clinicaltrials.gov/study/NCT05812326

[B26] Exploratory clinical trial on the safety, efficacy, and pharmacokinetics of XKDCT086 (iPD-1-Claudin18.2-CAR-T) in claudin 18.2 positive advanced solid malignant tumors: a Single center, Single arm, Dose-increasing Trial (2023a). Available at: https://clinicaltrials.gov/study/NCT05952375

[B27] Exploratory clinical trial on the safety, tolerability, efficacy, and pharmacokinetics of XKDCT080 in GCC-positive recurrent or refractory solid tumors with Single center, Single arm, and dose escalation.(2023b). Available at: https://clinicaltrials.gov/study/NCT05875402

[B28] Exploratory study of MSLN-CAR T cells secreting PD1/CTLA-4 nanoantibody for the treatment of advanced solid tumors.(2024). Available at: https://clinicaltrials.gov/study/NCT06248697

[B31] Exploratory study on the treatment of advanced solid tumor with non-viral vector multi-targeted autosecretory multifunctional antibody CAR-T cell injection (2024). Available at: https://clinicaltrials.gov/study/NCT06327997

[B32] An exploratory study on the treatment of advanced solid tumors by secretory PD1 nanoantibody targeting mesothelin fast CAR T cells. 2024 Available at: https://clinicaltrials.gov/study/NCT06249256

[B33] Ferreros P. Trapero I. (2022). Interleukin inhibitors in cytokine release syndrome and neurotoxicity secondary to CAR-T therapy. Diseases 10 (3), 41. 10.3390/diseases10030041 35892735 PMC9326641

[B34] Fih A. Arm S. (2022). Open label study to evaluate the safety, tolerability, pharmacokinetics and preliminary antitumor activity of autologous CAR T cells targeting BT-001. Patients Adv. Solid Tumors. Available at: https://clinicaltrials.gov/study/NCT05621486.

[B36] Freyer C. W. Porter D. L. (2020). Cytokine release syndrome and neurotoxicity following CAR T-cell therapy for hematologic malignancies. J. Allergy Clin. Immunol. 146 (5), 940–948. 10.1016/j.jaci.2020.07.025 32771558

[B37] FTiH (2023). “Phase 1 investigator-initiated trial (IIT) to evaluate the safety, feasibility,” in Cellular kinetics, and preliminary antitumor activity of AZD6422 in adult participants with advanced or metastatic CLDN18. 2+ GI Tumors. Available at: https://clinicaltrials.gov/study/NCT05981235.

[B38] Gardner R. A. Ceppi F. Rivers J. Annesley C. Summers C. Taraseviciute A. (2019). Preemptive mitigation of CD19 CAR T-cell cytokine release syndrome without attenuation of antileukemic efficacy. Blood 134 (24), 2149–2158. 10.1182/blood.2019001463 31697826 PMC6908832

[B39] Gazeau N. Liang E. C. Wu Q. Voutsinas J. M. Barba P. Iacoboni G. (2023). Anakinra for refractory cytokine release syndrome or immune effector cell-associated neurotoxicity syndrome after chimeric antigen receptor T cell therapy. Transplant. Cell. Ther. 29 (7), 430–437. 10.1016/j.jtct.2023.04.001 37031746 PMC10330552

[B40] GD2/PSMA Bi-specific CAR-T cells for the treatment of GD2 and PSMA positive solid tumors (2022). Available at: https://clinicaltrials.gov/study/NCT05437315

[B41] Grosskopf A. K. Labanieh L. Klysz D. D. Roth G. A. Xu P. Adebowale O. (2022). Delivery of CAR-T cells in a transient injectable stimulatory hydrogel niche improves treatment of solid tumors. Sci. Adv. 8 (14), eabn8264. 10.1126/sciadv.abn8264 35394838 PMC8993118

[B42] Kalos M. Levine B. L. Porter D. L. Katz S. Grupp S. A. Bagg A. (2011). T cells with chimeric antigen receptors have potent antitumor effects and can establish memory in patients with advanced leukemia. Sci. Transl. Med. 3 (95), 95ra73. 10.1126/scitranslmed.3002842 PMC3393096 21832238

[B43] Korell F. Berger T. R. Maus M. V. (2022). Understanding CAR T cell-tumor interactions: paving the way for successful clinical outcomes. Med 3 (8), 538–564. 10.1016/j.medj.2022.05.001 35963235

[B44] Labanieh L. Majzner R. G. Klysz D. Sotillo E. Fisher C. J. Vilches-Moure J. G. (2022). Enhanced safety and efficacy of protease-regulated CAR-T cell receptors. Cell 185 (10), 1745–1763.e22. 10.1016/j.cell.2022.03.041 35483375 PMC9467936

[B45] Larkin (2021). FDA emergency use authorized treatment protocol of DeltaRex-G for severe covid-19 [IND# 27185]. Eur. J. Respir. Med. 3 (2). 10.31488/EJRM.119

[B46] Liu S. Chawla S. P. Bruckner H. Morse M. A. Federman N. Srikureja A. (2021). Long term survival following DeltaRex-G/DeltaVax tumor-targeted gene therapy for advanced chemotherapy-resistant malignancies: an academic milestone. Clin. Oncol. 6, 1807. 10.25107/2474-1663.1807

[B47] Liu S. Deng B. Yin Z. Pan J. Lin Y. Ling Z. (2020). Corticosteroids do not influence the efficacy and kinetics of CAR-T cells for B-cell acute lymphoblastic leukemia. Blood Cancer J. 10 (2), 15. 10.1038/s41408-020-0280-y 32029707 PMC7005173

[B48] Maude S. L. Barrett D. Teachey D. T. Grupp S. A. (2014). Managing cytokine release syndrome associated with novel T cell-engaging therapies. Cancer J. 20 (2), 119–122. 10.1097/PPO.0000000000000035 24667956 PMC4119809

[B49] Melero I. Castanon E. Alvarez M. Champiat S. Marabelle A. (2021). Intratumoural administration and tumour tissue targeting of cancer immunotherapies. Nat. Rev. Clin. Oncol. 18 (9), 558–576. 10.1038/s41571-021-00507-y 34006998 PMC8130796

[B50] Mesothelin/GPC3/GUCY2C targeted CAR-T for immunotherapy of pancreatic cancer: phase I clinical trial. (2023). Available at: https://clinicaltrials.gov/study/NCT05779917

[B51] Morse M. Chawla S. Wong T. Bruckner H. Hall F. Gordon E. (2021). Tumor protein p53 mutation in archived tumor samples from a 12-year survivor of stage 4 pancreatic ductal adenocarcinoma may predict long-term survival with DeltaRex-G: a case report and literature review. Mol. Clin. Oncol. 15 (3), 186. 10.3892/mco.2021.2348 34277005 PMC8278409

[B52] Open An (2020). Multicenter, phase ib/II study to evaluate the efficacy, safety and pharmacokinetics of CT041 autologous CAR T-cell injection in patients with advanced gastric/gastroesophageal junction adenocarcinoma and. Pancreat. Cancer. Available at: https://clinicaltrials.gov/study/NCT04581473.

[B53] Open-Label An (2022). Single-arm, dose-exploration study to evaluate the safety, tolerability, preliminary efficacy and pharmacokinetics of CT048 in subjects with advanced solid tumors. Available at: https://clinicaltrials.gov/study/NCT05393986.

[B58] Phase I. (2022). II study of pan-T booster Co-expressing MSLN CAR T cell therapy in advanced/metastatic solid tumors. Available at: https://clinicaltrials.gov/study/NCT05693844.

[B62] Phase I clinical study of chimeric antigen receptor T cells (C-13-60) in the treatment of carcinoembryonic antigen (CEA) positive advanced malignant solid tumors (2023). Available at: https://clinicaltrials.gov/study/NCT06043466

[B65] Phase I study of autologous T Lymphocytes expressing GD2-specific chimeric antigen and constitutively active IL-7 receptors for the treatment of patients with relapsed or refractory neuroblastoma and other GD2 positive solid cancers(GAIL-N) (2018). Available at: https://clinicaltrials.gov/study/NCT03635632

[B67] Phase I/II study of anti-GD2 chimeric antigen receptor-expressing T cells in pediatric patients affected by high risk and/or relapsed/refractory neuroblastoma or other GD2-positive solid tumors (2017). Available at: https://clinicaltrials.gov/study/NCT03373097

[B68] Phase I/II study of CD70 targeted CAR-T cell treatment in CD70 positive advanced/metastatic solid tumors (2023). Available at: https://clinicaltrials.gov/study/NCT05947487

[B69] Phase I study of EGFR, Targeted tgfβr-KO CAR T cells in the treatment of previously treated advanced EGFR-positive solid tumors (2021). Available at: https://clinicaltrials.gov/study/NCT04976218

[B70] Qin Y. Xu G. (2022). Enhancing CAR T-cell therapies against solid tumors: mechanisms and reversion of resistance. Front. Immunol. 13, 1053120. 10.3389/fimmu.2022.1053120 36569859 PMC9773088

[B74] Sermer D. Brentjens R. (2019). CAR T‐cell therapy: full speed ahead. Hematol. Oncol. 37 (S1), 95–100. 10.1002/hon.2591 31187533

[B76] Single-center A. (2022a). “Open-label, single-arm clinical study of the safety and efficacy of KD-025 CAR-T therapy,” in Advanced NKG2DL+ solid tumors. Available at: https://clinicaltrials.gov/study/NCT05382377.

[B77] Single-center A. (2022b). Open-label, single-arm clinical study of the safety and efficacy of KD-496 CAR-T therapy in advanced nkg2dl+/CLDN18. 2+ Solid Tumors. Available at: https://clinicaltrials.gov/study/NCT05583201.

[B79] T cells armed with chimeric antigen receptor recognizing EpCAM for patients with advanced solid tumors (2016). Available at: https://clinicaltrials.gov/study/NCT02915445

[B80] Ventin M. Cattaneo G. Maggs L. Arya S. Wang X. Ferrone C. R. (2024). Implications of high tumor burden on chimeric antigen receptor T-cell immunotherapy: a review. JAMA Oncol. 10 (1), 115–121. 10.1001/jamaoncol.2023.4504 37943567

[B81] Xia H. Li X. Gao W. Fu X. Fang R. H. Zhang L. (2018). Tissue repair and regeneration with endogenous stem cells. Nat. Rev. Mater 3 (7), 174–193. 10.1038/s41578-018-0027-6

[B82] Zhu I. Liu R. Garcia J. M. Hyrenius-Wittsten A. Piraner D. I. Alavi J. (2022). Modular design of synthetic receptors for programmed gene regulation in cell therapies. Cell 185 (8), 1431–1443.e16. 10.1016/j.cell.2022.03.023 35427499 PMC9108009

